# Potential of Sea Urchin *Mesocentrotus nudus* as a Target Catch Species in the Pacific Ocean off Eastern Hokkaido, Japan

**DOI:** 10.3390/ani14121740

**Published:** 2024-06-08

**Authors:** Satomi Takagi, Natsuki Hasegawa

**Affiliations:** Kushiro Field Station, Fisheries Resources Institute, Japan Fisheries Research and Education Agency, Kushiro 085-0802, Hokkaido, Japan

**Keywords:** sea urchin, distribution, *Mesocentrotus nudus*, maturation, global warming

## Abstract

**Simple Summary:**

Ocean warming has led to shifts in species distributions. The sea urchin *Mesocentrotus nudus* is commercially harvested in northern Japan. Although limited scientific reports on the presence of this species in Hokkaido are available from Cape Soya to Cape Erimo along the coast of the Sea of Japan, statistical data from catches indicate that it has extended its range to the Sea of Okhotsk and Pacific Ocean. In 2021, harmful algal blooms (HABs) occurred along the Pacific coast of Hokkaido, and massive die-offs of marine organisms, including *M. nudus,* were reported. This study aimed to redefine the presence of *M. nudus* in the Pacific Ocean off Hokkaido after the HABs. Field surveys were conducted, and *M. nudus* was confirmed in Akkeshi, the site farthest from Cape Soya among the areas where irregular catches of *M. nudus* were recorded in eastern Hokkaido, in 2023. All sea urchins were >6 years of age, indicating that they survived the HABs. The size and developmental stages of the ovaries and testes of collected individuals suggest the reproductive cycle of *M. nudus* in Akkeshi would synchronize with that of the specimens off Wakkanai, Cape Soya. Warming trends may lead to population increases in the future.

**Abstract:**

Scientific reports on the distribution of *Mesocentrotus nudus* in Hokkaido are limited from Cape Soya to Cape Erimo along the coast of the Sea of Japan; however, fishery statistics show that its distribution has extended to the Sea of Okhotsk and Pacific Ocean off Hokkaido. In 2021, large-scale harmful algal blooms (HABs) occurred in the Pacific Ocean off eastern Hokkaido, resulting in the massive die-off of marine organisms, including *M. nudus*. This study aimed to redefine the distribution of *M. nudus* in the Pacific Ocean off eastern Hokkaido after the HABs. Field surveys were conducted in July, August, and December 2023 in Akkeshi, the site farthest from Cape Soya among the areas where irregular catches of *M. nudus* have been recorded in eastern Hokkaido, and the distribution of this species was confirmed in August and December. All sea urchins collected were >6 years of age, indicating that they survived the HABs. High gonad indices and spermatozoa-filled gonads were observed in the sea urchins collected in December, suggesting that the reproductive cycle of *M. nudus* in Akkeshi may be close to that observed in specimens off Wakkanai, Cape Soya. Warming trends may cause population increases in the future.

## 1. Introduction

**Figure 1:**
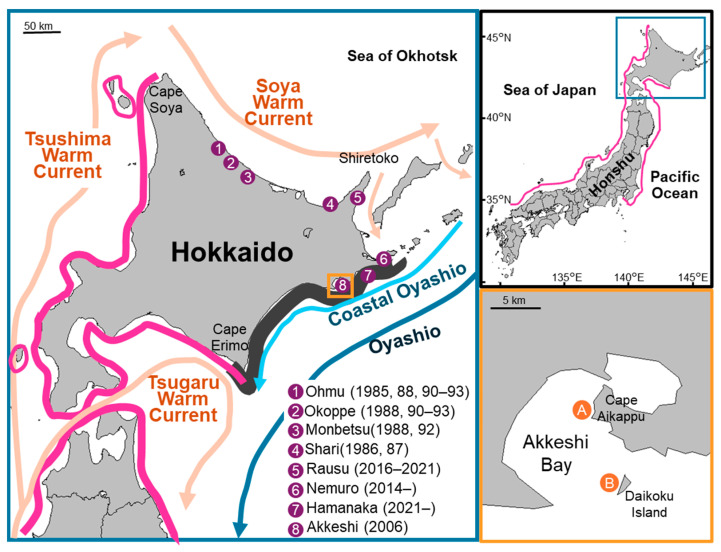
Distribution of *Mesocentrotus nudus* in Japan. Bold pink lines indicate the distribution of *M. nudus* based on previous studies. Constant catches of *M. nudus* have been conducted from Cape Soya to Cape Erime along the western coast of Hokkaido since at least 1985. Purple dots with numbers indicate towns in Hokkaido that have recorded irregular catches of *M*. *nudus*. Numbers in parentheses indicate the year in which these catches were recorded. Arrows indicate the schematic of surface ocean currents around Hokkaido during summer–autumn seasons. Orange dots marked A and B indicate the study sites. The dark grey shaded area roughly indicates coastal waters where fisheries experienced devastating damage attributed to harmful algal blooms in 2021.

Ocean warming has led to shifts in the distribution of marine ectotherm species, such as in poleward range extensions [1,2]. For example, poleward extensions have been reported for the sea urchins *Hemicentrotus pulcherrimus* [3] and *Heliocidaris crassispina* [4] in the Sea of Japan and *Centrostephanus rodgersii* in eastern Australia [5,6] and New Zealand [7]. The sea urchin *Mesocentrotus nudus* is commercially harvested in northern Japan. Together with *Strongylocentrotus intermedius*, *M. nudus* accounts for more than two-thirds of the total quantity of sea urchins landed in Japan [8]. Furthermore, the price per wooden tray of the gonads of *M. nudus* is among the highest worldwide for sea urchins [8]. The distribution range was considered to be from Dalian in China to Primorsky Krai in Russia on the Eurasian continent [9,10,11,12]. In Japan, the range was along the Pacific Ocean from Sagami Bay to Cape Erimo, Hokkaido, and the Sea of Japan from Omi Island, Yamaguchi to Cape Soya, Hokkaido [9,10,11,12] (Figure 1). Constant catches of *M. nudus* have been conducted from Cape Soya to Cape Erimo along the western coast of Hokkaido since at least 1985, when the Hokkaido government began recording data on sea urchin landings by species [13]. Meanwhile, the extension of *M. nudus* distributions into colder water has also been documented, although the specific range of the extension was not mentioned [14]. Inconsistent catches of *M. nudus* in the Sea of Okhotsk and Pacific Ocean around Hokkaido have been recorded (Figure 1) [13], suggesting that the distribution has reached the Pacific Ocean. However, to the best of our knowledge, scientific reports are lacking on wild *M. nudus* in the Pacific Ocean off Hokkaido. Around Hokkaido, the Tsushima Warm Current flows from south to north in the Sea of Japan and extends to the Sea of Okhotsk and Pacific Ocean as the Soya Warm Current and Coastal Oyashio Current, respectively [15,16,17]. In the Pacific Ocean, the cold Oyashio flows from north to south along the continental slope off eastern Hokkaido [18]. Therefore, the water temperature in the Pacific Ocean off eastern Hokkaido is particularly low (Figure S1). The surface water temperature off Kushiro in eastern Hokkaido increased by 1.66 °C/century, which is higher than the average for the entirety of Japan, and the annual average temperature off Kushiro has continuously increased since the 2000s [19]. Of the areas where irregular catches of *M. nudus* have been recorded (Ohmu, Okoppe, Monbetsu, Shari, Rausu, Nemuro, Hamanaka, and Akkeshi), the site farthest from Cape Soya is Akkeshi, for which catches of *M. nudus* were only reported in 2006. In addition, unprecedented large-scale harmful algal blooms (HABs) dominated by dinoflagellate *Karenia selliformis* occurred along the coast of the Pacific Ocean off eastern Hokkaido from September to November 2021 [20]. A massive die-off of marine organisms such as *S. intermedius*, the target species for catch in the region, was reported. We confirmed the collection of dead wild *M. nudus* off Hamanaka (Kushiro Fisheries Technical Guidance Office, unpublished data). Additionally, 40% mortality rates of adult *M. nudus* were observed at the experimental aquariums of Kushiro Field Station, Japan Fisheries Research and Education Agency (42.9499° N, 144.4429° E) [21]. Hokkaido fishery statistics show that *S. intermedius* was still caught in 2021 and 2022 in the area damaged by the HABs. However, the volume drastically declined during this period, with the gonadal weight of the Akkeshi catch decreasing from 9936 kg in 2020 to 2951 kg in 2021 and 2294 kg in 2022 [13]. It is unclear whether *M. nudus* still inhabits Akkeshi waters.

The present study aimed to redefine the distribution of *M. nudus* in eastern Hokkaido after the HABs that occurred in 2021. We conducted field surveys in Akkeshi Bay in 2023 and confirmed the presence of *M. nudus*. In addition, the habitat and biological information of the sea urchins were compared with those from the main distribution areas based on previous studies. To the best of our knowledge, the present study represents the first documented scientific information on *M. nudus* along the Pacific coast of eastern Hokkaido.

## 2. Materials and Methods

Field surveys were conducted at two rocky shores in Akkeshi Bay: Site A off Cape Aikappu on 19 July and 15 December 2023 and Site B off Daikoku Island on 21 August 2023 (Figure 1). Transect lines of 150 m and 200 m from shore to offshore were placed at sites A and B, respectively. The starting points near shore were covered with surfgrass (*Phyllospadix iwatensis*) at a density of >90% (Site A, 43.0194° N, 144.9351° E, at a depth of 0.9 m; Site B, 42.9542° N, 144.8667° E, at a depth of 1.8 m). Three 1 m^2^ quadrats were placed at locations 30, 50, 80, and 100 m from the starting point in July, at locations 50, 100, 150, and 200 m from the starting point in August, and at locations 50, 100, and 150 m from the starting point in December. For each quadrat, the divers recorded on waterproof paper the depth, substrate, understory species, percentage cover of canopy-forming species, and number of sea urchins, Laminariales, and Fucales. *Saccharina japonica* var. *diabolica* is a biennial species; thus, specimens at 1st and 2nd years of ages were recorded separately when observed in the quadrats. Then, the divers actively searched within 20 m of the transects for sea urchins, counted the number of individuals by species, and collected all specimens of *M. nudus* they found. The depths and substrates where *M. nudus* was collected were recorded. More than five individuals of each species of Laminariales and Fucales were collected, and the average dry weight was used to calculate the biomass. The biomass of sea sorrel *Desmarestia viridis*, green laver *Ulva* spp., and other small specimens of red and brown algae were calculated using the dry weight and cover percentage in 0.25 m^2^ quadrats placed near the transect lines. 

The collected *M. nudus* were placed in a cool box containing moist polyurethane mats immersed in seawater and transported to Kushiro Field Station within 2 h and kept in running filtered seawater. On the following day, the test diameter (accurate to within 0.01 mm) and body wet weight (accurate to within 0.1 g) of sea urchins were measured using vernier calipers and an electronic balance, respectively, and sea urchins were dissected. Gonad wet weight (accurate to within 0.01 g) was measured after excess water was blotted off, and the gonad index (gonad wet weight × 100/body wet weight) was calculated. A section of each sampled gonad was fixed in Davidson’s solution, dehydrated, and embedded in paraffin wax. Sections with a thickness of 10 µm were prepared, dewaxed, stained with hematoxylin and eosin, and observed under a light microscope (BH-2, Olympus Corporation, Tokyo, Japan). The gonadal maturities of the animals were classified into five stages as described by Fuji [22] with minor modifications [23]: recovering (stage 1, before gametogenesis), growing (stage 2, early gametogenesis), premature (stage 3, mid-gametogenesis), mature (stage 4, late gametogenesis), and spent (stage 5, after spawning). Age was determined by counting the number of black bands formed, as seen on charred genital plates [24,25]. To compare the water temperature at the study sites with the main distribution area of *M. nudus*, the water temperature at Kushiro near Akkeshi during 1984–1993 and 2014–2023 was calculated using the daily water temperature from Japan Fisheries Research and Education Agency [26], whereas that at Yoichi in western Hokkaido during 1984–1993 and at Wakkanai in Cape Soya during 1986–1993 (data collection was started in 1986) was calculated using the daily water temperature from Hokkaido Aquaculture Promotion Corporation [27]. On days for which data were not available, the average of the previous and following days was used as the temperature for that day.

## 3. Results

Two Laminariales (*S. japonica* var. *diabolica*, *Costaria costata*) and one Fucales (*Stephanocystis hakodatensis*) species were distributed at Site A in July (Table 1). The dominant species at a distance of 30 m (1.8 m depth) from the starting points was *S. japonica* var. *diabolica*, and the coverage was >90%. The biomass of Laminariales decreased with increasing depth (Figure S2). The substrate was comprised of boulders and rocks throughout the transect line, and *D. viridis*, *Ulva* spp., and articulated coralline algae were observed as understory species. In particular, *D. viridis* dominated at a distance of 80 m and 100 m (at depths of 3.5 m and 2.6 m, respectively), and covered 96.7 ± 5.8% and 71.8 ± 22.5% of the substrate at these distances, respectively. The density of *S. intermedius* at 50–100 m was higher than that observed at 30 m. A total of 41 *S. intermedius* specimens were identified during the 57 min search, although no *M. nudus* were observed (Table S1). In December, *S. japonica* var. *diabolica* at 1st year of age, and *C. costata* were observed at 50 m; however, the biomass of these kelps was quite low compared with that observed in July. The articulated coralline algae covered 10.0 ± 0.0%, 10.0 ± 0.0%, and 16.7 ± 5.8% of the substrates at 50, 100, and 150 m, respectively. Small red algae, including *Tichocarpus crinitus* and *Ptilota* spp., and juvenile *S. hakodatensis,* were observed at 150 m. The abundance of *S. intermedius* at 100 and 150 m was higher than that at 50 m. Two specimens of *M. nudus* and 43 specimens of *S. intermedius* were identified during the 20 min search (Table S1). *M. nudus* was observed near the transect line at 50 m, and the substrates were boulders and rocks with articulated coralline algae.

**Figure 2:**
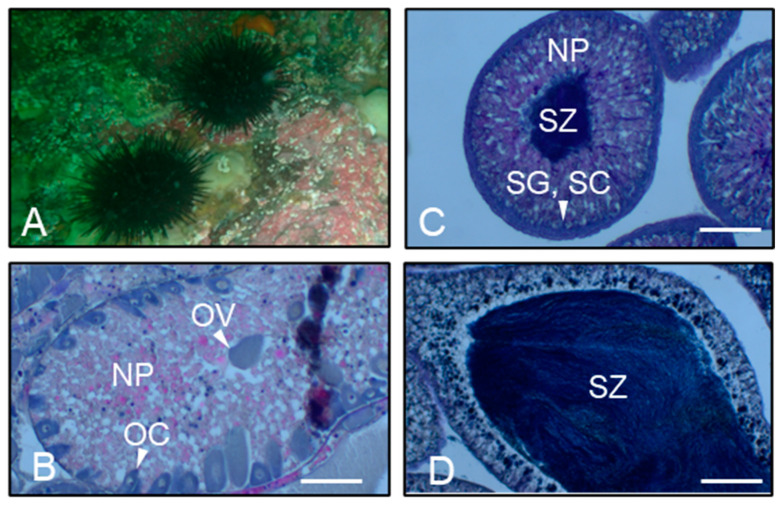
Photo of *Mesocentrotus nudus* observed off Daikoku Island in Akkeshi Bay (**A**) and histology of *M. nudus* gonads (**B**–**D**); (**B**), ovaries at stage III (pre-mature); (**C**), testes at stage III (pre-mature); and (**D**), testes at stage V observed in December. The testes were filled with sperm, but clusters of spermatogonia and spermatocytes were not observed, and phagocytosis by nutrient phagocytes started. NP, nutrient phagocyte; OC, oocyte; SG, spermatogonium; OV, ripe ova; SZ, spermatozoa. Scale bars: 200 μm.

At Site B, Laminariales kelp dominated at a distance of 50–150 m along the survey line (at a depth of 2.5–3.6 m), and crustose corallines covered the substrates at 150 m and 200 m (at depths of 3.6 m and 7.1 m, respectively). Dominant kelp species were *S. japonica* var. *diabolica* at 50 m and 100 m and *Saccharina coriacea* at 150 m. *S. japonica* var. *diabolica* at 1st year of age was observed at 2.5 m depths. Small red algae *Neodilsea yendoana, T. crinitus,* and *Ptilota* spp. were observed as understory species. *S. intermedius* was observed at 50 m and 200 m, and the density was low compared with that at Site A. Seven specimens of *M. nudus* and three specimens of *S. intermedius* were identified at an undulated boulder covered with crustose corallines at depths of 7.0 m outside the transect line during the 7 min search (Table S1; Figure 2A). 

Table 2 shows the test diameter, body weight, gonad weight, gonad indices, and age of the collected *M. nudus*. Test diameters and gonad indices of sea urchins were greater than the minimum size for commercial landings (≥50 mm test diameter; gonad index of 18 [28]). All individuals were 6–9 years of age, indicating they survived the HABs that occurred in 2021. The gonads of one male sea urchin from Site A and those of four males and three females from Site B were at stage 3; nutrient phagocytes (NPs) were partly replaced with ripe ova or spermatozoa, and numerous developing oocytes or clusters of spermatogonia and spermatocytes were present at the periphery of the acini (Figure 2B,C). The gonadal lumina of one male from Site A were filled with spermatozoa, whereas clusters of spermatogonia and spermatocytes, which should be observed in testes at stage 4, were not observed. Instead, phagocytosis by NPs had started (Figure 2D). Thus, we determined these gonads to be at stage V. 

Figure S1 shows the seasonal changes in water temperature at Kushiro, Wakkanai, and Yoichi. During 1984–1993, the lowest temperatures at Kushiro (–0.49 °C) and Wakkanai (0.01 °C) were similar, but the highest temperature at Kushiro (16.02 °C) was lower than that at Wakkanai (21.73 °C). The temperature at Yoichi was approximately 5 °C higher than that at Kushiro throughout any given year. The temperature at Yoichi and Wakkanai reached a peak in mid-August, while that at Kushiro reached a peak in mid-September. The highest temperature at Kushiro increased to 17.38 °C in 2014–2023 and reached 20.63 °C in 2023. The lowest temperature at Kushiro showed limited changes over the last 40 years. 

## 4. Discussion

This study confirmed the presence of *M. nudus* in Akkeshi Bay in eastern Hokkaido; however, the number of collected individuals was quite small. The habitat of *M. nudus* at Site A was boulders and rocks, while that at Site B was dominated by crustose corallines, which is similar to the habitats of *M. nudus* in southwestern Hokkaido and Tohoku, as described by Agatsuma [29]. The gonad size of *M. nudus* in the present study was greater than the minimum size for commercial landings (gonad index of 18 [26]). *M. nudus* is an herbivorous species and prefers Laminariales kelp (reviewed by Agatsuma [29]). The gonad size of *M. nudus* is affected by the type and abundance of algae in its habitat; the gonad indices of *M. nudus* in crustose coralline-dominated barren were low because of the low food availability [12,30,31]. Laminariales kelp beds formed at Sites A and B in summer. Therefore, the high gonad indices of *M. nudus* observed in the present study would result from the food supply from nearby kelp beds. 

The gonad maturation stages of *M. nudus* change from stages II to IV from June to August and to stage V during September and October in southern Hokkaido and Tohoku [12,32,33,34]. Sugimoto et al. [32] investigated the annual reproductive cycle of *M. nudus* at three sites (Teuri Island, Rebun Island, and Wakkanai) off Cape Soya, northern Hokkaido during 1977–1978, and concluded that maturation of *M. nudus* starts during July–August, and spawning season lasts from September to October. Meanwhile, gonad indices of *M. nudus* in Wakkanai maintained >17, after the decline of gonad indices occurred during September and October, and gonads filled with relict spermatozoa were observed during winter to spring [32]. High gonad indices and testes filled with spermatozoa in December in the present study were similar to the gonadal characteristics of *M. nudus* in Wakkanai. Furthermore, the highest and lowest water temperatures observed at Kushiro in 2023 were similar to those seen at Wakkanai during 1986–1993 (Figure S1). Because large variations were not observed in the maximum temperatures in Wakkanai during 1986–1993, Yoichi during 1984–1993, and Kushiro in 2023, the temperature in winter or high-water temperature periods in summer might have influenced the continuous maturation until winter and/or largely reliction of spermatozoa in December. 

A high density of >10 individuals of *M. nudus* in a 1 m^2^ quadrat was observed in crustose coralline algal communities in southern Hokkaido and Tohoku, as reviewed by Agatsuma [29]. In the present study, only two and seven individuals were found at Sites A and B, respectively, during the surveys, and the density of *M. nudus* at both sites was considerably lower than that of *S. intermedius*, although it needs to be taken into account that juvenile *S. intermedius* is reseeded in this region [14]. The larval stage of *M. nudus* off Shakotan Peninsula in the Sea of Japan is estimated to occur over 1 to 2 months [29]. The optimal temperature for embryos to develop into pluteus larvae of *M. nudus* and *S. intermedius* is estimated to be 10–22 °C and 4–20 °C, respectively, and the rate of development decreases with a decrease in temperature [9]. The larval period of plutei of *M. nudus* is 14–18 days at 22.2 °C, which is shorter than the period of 19–22 days at 24.9 °C and 18–25 days at 19.4 °C [35]. The density of *M. nudus* at 1 year of age was high when the water temperature of the previous September was 20–23 °C [12]. The water temperature at Kushiro rarely reached 20 °C, and since 1984, this temperature was only achieved in 1999, 2010, 2016, 2020, 2021, and 2023 [26]. These findings indicate that the temperature off Kushiro has not been suitable for the early development and settlement of *M. nudus*, and the larval stage would be longer than that in the main distribution area, resulting in a low population. However, warming trends would promote an increase in the population of *M. nudus* in this location in the future.

The northward extension of *H. crassispina* in the Sea of Japan has been reported [4]; however, in its new habitat, larval supply from the main distribution area and low possibility of self-reproduction are suggested because of the asynchronous spawning between the genders [4,36]. The present study could not clarify whether *M. nudus* in Akkeshi Bay was self-reproduced or the larvae were supplied from northern Hokkaido because of limitations in the sample size. However, the continuous maturation until winter and/or occurrence of relict spermatozoa in December in the present study suggests that spawning problems may occur in eastern Hokkaido. Annual investigations of gonad development with a larger number of samples from the study site are required to shed further light on this issue. 

Catches of *S. intermedius* in Hokkaido sharply decreased during 1988–1991 and then stagnated before gradually declining in the 2000s [37]. Gouda and Agatsuma [38] reported that increases in water temperature delay maturation and spawning, leading to low recruitment. Marine heatwaves have become more frequent in the northeastern Pacific since 2010 [39], and the warmest global ocean surface temperature was recorded in 2023 [40]. Global warming is considered one of the factors underlying the increased risk of HABs. The frequency and geographic distribution of HABs have increased in recent decades [41]. In 2021, unprecedented large-scale HABs occurred off southeastern Hokkaido, which has rarely experienced HABs because the area is facing the North Pacific Ocean. Approximately one-month prior, marine heatwaves occurred in the northeastern Pacific, and they likely preconditioned and accelerated the blooms [42]. All specimens of *M. nudus* collected in the present study were ≥6 years of age, indicating that the sea urchins survived the HABs that occurred in 2021. Catches of *S. intermedius* in the Pacific Ocean off eastern Hokkaido drastically decreased after the HABs [13]. *M. nudus*, with its tolerance for high-temperature waters, has the potential to be an important target catch species in the future.

## 5. Conclusions

Scientific reports on the distribution of *M. nudus* in Hokkaido, Japan have been limited from Cape Soya to Cape Erimo along the coast of the Sea of Japan. The present study confirmed the distribution of *M. nudus* in Akkeshi Bay in 2023 and, to the best of our knowledge, is the first to document scientific information on *M. nudus* in the Pacific Ocean off eastern Hokkaido. All *M. nudus* collected were 6–9 years of age, indicating that they survived the HABs that occurred in 2021. Although there was a limitation in sample size, histological observation on the gonads of *M. nudus* showed the continuous maturation until winter and/or occurrence of relict spermatozoa in testes in December, suggesting that the reproductive cycle of *M. nudus* in Akkeshi may be close to that observed in specimens off Wakkanai, Cape Soya, northern Hokkaido. Investigation on the annual reproductive cycle of *M. nudus* in the Pacific Ocean off eastern Hokkaido with a larger number of samples are required to clarify the possibility of the self-reproduction in this area. Water temperature off eastern Hokkaido is increasing and approaching the temperature of the main distribution area of *M. nudus*. Warming trends may lead to an increase in *M. nudus* populations, and *M. nudus* has the potential to be an important target catch species in the Pacific coast of eastern Hokkaido in the future. 

## Figures and Tables

**Table 1:** Density of Laminariales, Fucales, and sea urchins along the transect lines (ind/m^2^; n = 3).

Site	Month	Distance from the Starting Point	Depth	*Saccharina japonica* var. *diabolica*		*Costaria costata*	*Saccharina coriacea*	*Agarum clathratum*	*Stephanocystis hakodatensis*	*Strongylocentrotus intermedius*
			m	1 y	2 y					
A	July	30 m	1.8 ± 0.0		5.7 ± 3.8				2.3 ± 4.0	0.3 ± 0.6
		50 m	2.4 ± 0.1			4.3 ± 5.1				4.7 ± 5.5
		80 m	3.5 ± 0.1			0.3 ± 0.6				3.7 ± 5.5
		100 m	2.6 ± 0.1	1.7 ± 2.1		2.0 ± 2.6				3.0 ± 3.0
	December	50 m	3.4 ± 0.1	0.3 ± 0.6		0.3 ± 0.6				1.3 ± 2.3
		100 m	3.3 ± 0.3							6.0 ± 3.0
		150 m	4.7 ± 0.3							5.0 ± 2.0
B	August	50 m	2.5 ± 0.1	50.0 ± 27.8	6.7 ± 2.9					0.3 ± 0.6
		100 m	2.5 ± 0.1	31.0 ± 32.9		1.7 ± 1.5	1.3 ± 1.5		1.7 ± 0.6	
		150 m	3.6 ± 0.1		2.5 ± 0.7		12.7 ± 3.8		0.3 ± 0.6	
		200 m	7.1 ± 0.4	0.7 ± 1.2	1.0 ± 1.7		1.0 ± 1.0	8.3 ± 5.7	0.3 ± 0.6	1.0 ± 1.0

**Table 2:** Test diameter, body weight, gonad weight, gonad indices, and age of *Mesocentrotus nudus* collected in Akkeshi Bay.

Site	Month	N	Test Diameter	Body Weight	Gonad Weight	Gonad Index	Age			
			mm	g	g		VI	VII	VIII	IX
A	December	2	74.31 ± 5.81	175.5 ± 23.9	32.66 ± 9.02	19.43 ± 2.63			1	1
B	August	7	66.32 ± 3.71	121.7 ± 14.0	25.73 ± 4.81	21.07 ± 2.49	5	2		

## Data Availability

The raw data supporting the conclusions of this article will be made available by the authors, without undue reservation.

## Supplementary Materials

The following supporting information can be downloaded at https://www.mdpi.com/article/10.3390/ani14121740/s1.

## References

[1] Parmesan C., Yohe G. (2003). A globally coherent fingerprint of climate change impacts across natural systems. Nature.

[2] Poloczanska E.S., Brown C.J., Sydeman W.J., Kiessling W., Schoeman D.S., Moore P.J., Brander K., Bruno J.F., Buckley L.B., Burrows M.T. (2013). Global imprint of climate change on marine life. Nat. Clim. Chang..

[3] Agatsuma Y., Hoshikawa H. (2007). Northward extension of geographic range of the sea urchin *Hemicentrotus pulcherrimus* in Hokkaido, Japan. J. Shellfish Res..

[4] Feng W., Nakabayashi N., Narita K., Inomata E., Aoki M.N., Agatsuma Y. (2019). Reproduction and population structure of the sea urchin *Heliocidaris crassispina* in its newly extended range: The Oga Peninsula in the Sea of Japan, northeastern Japan. PLoS ONE.

[5] Andrew N.L., Byrne M., Lawrence J.M. (2001). The ecology of *Centrostephanus rodgersii*. Edible Sea Urchins: Biology and Ecology.

[6] Johnson C.R., Ling S.D., Ross D.J., Shepherd S., Miller K.J. (2005). Establishment of the Long-Spined Sea Urchin (Centrostephanus rodgersii) in Tasmania: First Assessment of Potential Threats to Fisheries.

[7] Pecorino D., Lamare M.D., Barker M.F. (2013). Reproduction of the Diadematidae sea urchin *Centrostephanus rodgersii* in a recently colonized area of northern New Zealand. Mar. Biol. Res..

[8] Unuma T., Brown N.P., Eddy S.D. (2015). Introduction: Sea urchin fisheries in Japan. Echinoderm Aquaculture.

[9] Fujisawa H., Shigei M. (1990). Correlation of embryonic temperature sensitivity of sea urchins with spawning season. J. Exp. Mar. Biol. Ecol..

[10] Kawamura K. (1993). Uni Zouyoushoku to Kakou, Ryutsu.

[11] Shigei M., Nishimura S. (1995). Echinozoa. Guide to Seashore Animals of Japan with Color Pictures and Keys.

[12] Agatsuma Y. (1997). Ecological studies on the population dynamics of the sea urchin *Strongylocentrotus nudus*. Sci. Rep. Hokkaido Fish. Exp. Stn..

[13] Hokkaido Fishery Annual Statistics, Fisheries and Forestry Divisions; Hokkaido Government: Sapporo, Japan.

[14] Agatsuma Y., Brown N.P., Eddy S.D. (2015). Reseeding of sea urchins in Japan. Echinoderm Aquaculture.

[15] Takizawa T. (1982). Characteristics of the soya warm Current in the Okhotsk sea. J. Oceanogr. Soc. Jpn..

[16] Uchimoto K., Mitsudera H., Ebuchi N., Miyazawa Y. (2007). Anticyclonic eddy caused by the Soya Warm Current in an Okhotsk OGCM. J. Oceanogr..

[17] Kuroda H., Taniuchi Y., Kasai H., Nakanowatari T., Setou T. (2021). Co-occurrence of marine extremes induced by tropical storms and an ocean eddy in summer 2016: Anomalous hydrographic conditions in the Pacific shelf waters off southeast Hokkaido, Japan. Atmosphere.

[18] Sakurai Y. (2007). An overview of the Oyashio ecosystem. Deep Sea Res. II.

[19] Japan Meteorological Agency Long-Term Trend of Average Surface Temperature off Kushiro. https://www.data.jma.go.jp/gmd/kaiyou/data/shindan/a_1/japan_warm/cfig/warm_area.html?area=J#title.

[20] Iwataki M., Lum W.M., Kuwata K., Takahashi K., Arima D., Kuribayashi T., Kosaka Y., Hasegawa N., Watanabe T., Shikata T. (2022). Morphological variation and phylogeny of *Karenia selliformis* (Gymnodiniales, Dinophyceae) in an intensive cold-water algal bloom in eastern Hokkaido, Japan. Harmful Algae.

[21] Hasegawa N., Watanabe T., Unuma T., Yokota T., Izumida D., Nakagawa T., Kurokawa T., Takagi S., Azumaya T., Taniuchi Y. (2022). Repeated reaching of the harmful algal bloom of *Karenia* spp. around the Pacific shoreline of Kushiro, eastern Hokkaido, Japan, during autumn 2021. Fish. Sci..

[22] Fuji A. (1960). Studies on the biology of the sea urchin: I. Superficial and histological gonadal changes in gametogenic process of two sea urchins, *Strongylocentrotus nudus* and *S. intermedius*. Bull. Fac. Fish Hokkaido Univ..

[23] Unuma T., Yokota Y., Matranga V., Smolenicka Z. (2002). Gonadal growth and its relationship to aquaculture in sea urchins. The Sea Urchin: From Basic Biology to Aquaculture.

[24] Jensen M. (1969). Age determination of Echinoids. Sarsia.

[25] Kawamura K. (1973). Studies in fisheries biology of sea urchin *Strongylocentrotus intermedius*. Sci. Rep. Hokkaido Fish. Exp. Stn..

[26] Water Temperature at Kushiro, Japan Fisheries Research and Education Agency. https://hnf.fra.affrc.go.jp/suion/suionjoho.html.

[27] Daily Water Temperature in Hokkaido, Hokkaido Aquaculture Promotion Corporation. https://www.saibai.or.jp/water_temp_info/daily/.

[28] Agatsuma Y. (1999). Gonadal growth of the sea urchin *Strongylocentrotus nudus*, from trophically poor coralline flats and fed excess kelp, *Laminaria religiosa*. Suisanzoshoku.

[29] Agatsuma Y., Lawrence J.M. (2020). *Mesocentortus* *nudus*. Sea Urchins: Biology and Ecology.

[30] Sano M., Omori M., Taniguchi K., Seki T. (2001). Age distribution of the sea urchin *Strongylocentrotus nudus* (A. Agassiz) in relation to algal zonation in a rocky coastal area on Oshika Peninsula, northern Japan. Fish. Sci..

[31] Takagi S., Murata Y., Inomata E., Endo H., Aoki M.N., Agatsuma Y. (2017). Improvement of gonad quality of the sea urchin *Mesocentrotus nudus* fed the kelp *Saccharina japonica* during offshore cage culture. Aquaculture.

[32] Sugimoto T., Tajima K., Tomita K. (1982). Reproductive cycles of the sea urchin, *Strongylocentrotus nudus*, on the northern coast of Hokkaido. Sci. Rep. Hokkaido Fish. Exp. Stn..

[33] Odagiri A., Asuke M., Sato K. (1984). Gonadal maturation of the sea urchin, *Strongylocentortus nudus*, inhibiting in the deep water off Okoppe, Aomori Prefecture. Sci. Rep. Aquac. Cen. Aomori Pref..

[34] Agatsuma Y., Motoya S., Sugawara Y. (1988). Reproductive cycle and food ingestion of the sea urchin, *Strongylocentrotus nudus* (A. Agassiz), in southern Hokkaido. I. Seasonal changes of gonad. Sci. Rep. Hokkaido Fish. Exp. Stn..

[35] Tsuchida K. Experiment on Settlement of Sea Urchins. *Annual Report of Iwate Prefectural Fisheries Experimental Station*, 1971, 1970, 60–61.

[36] Feng W., Nakabayashi N., Inomata E., Aoki M.N., Agatsuma Y. (2021). Sexually unbalanced gonad development and nutrition of the newly range-extended sea urchin *Heliocidaris crassispina* in the northeastern Honshu, Japan. Estuar. Coast. Shelf Sci..

[37] Agatsuma Y., Lawrence J.M. (2020). Stock enhancement of regular sea urchins. Sea Urchins: Biology and Ecology.

[38] Gouda H., Agatsuma Y. (2020). Effect of high temperature on gametogenesis of the sea urchin *Strongylocentrotus intermedius* in the Sea of Japan, northern Hokkaido, Japan. J. Exp. Mar. Biol. Ecol..

[39] Miyama T., Minobe S., Goto H. (2021). Marine heatwave of sea surface temperature of the Oyashio region in summer in 2010–2016. Front. Mar. Sci..

[40] NOOA (2023). Earth Had Its Warmest July on Record; Fourth Consecutive Month of Record-High Global Ocean Surface Temperature, Assessing the Global Climate in July 2023. https://www.ncei.noaa.gov/news/global-climate-202307.

[41] Fu F.X., Tatters A.O., Hutchins D.A. (2012). Global change and the future of harmful algal blooms in the ocean. Mar. Ecol. Prog. Ser..

[42] Takagi S., Kuroda H., Hasegawa N., Watanabe T., Unuma T., Taniuchi Y., Yokota T., Izumida D., Nakagawa T., Kurokawa T. (2022). Controlling factors of large-scale harmful algal blooms with *Karenia selliformis* after record-breaking marine heatwaves. Front. Mar. Sci..

