# Supplementary material for: Potential of Sea Urchin Mesocentrotus nudus as a Target Catch Species in the Pacific Ocean off Eastern Hokkaido, Japan
Source: Animals (Basel). 2024 Jun 8;14(12):1740. doi: 10.3390/ani14121740 (PMC11201216; doi:10.3390/ani14121740)
Supplement: Supplementary file 1 [file animals-14-01740-s001.zip › animals-2996372-supplementary.pdf]

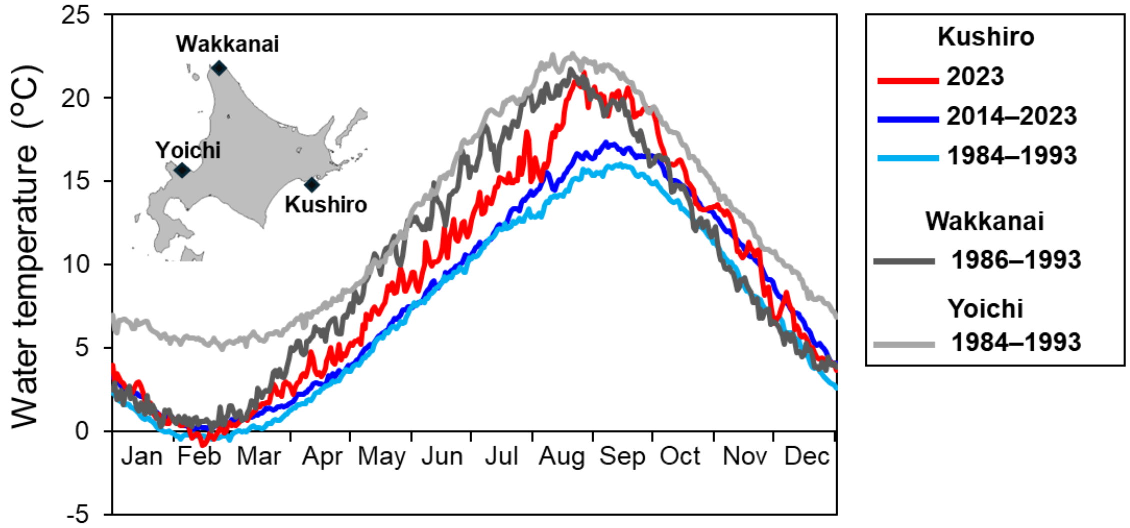

Figure S1. Annual changes of water temperature at Kushiro, Wakkanai and Yoichi. Data at Kushiro was from Japan Fisheries Research and Education Agency (http://hnf.fra.affrc.go.jp/suion/suionjoho.html ; accessed on March 25, 2024), and that at Wakkanai and Yoichi were from Hokkaido Aquaculture Promotion Corporation (https://www.saibai.or.jp/water_temp_info/daily/; accessed on March 25, 2024).

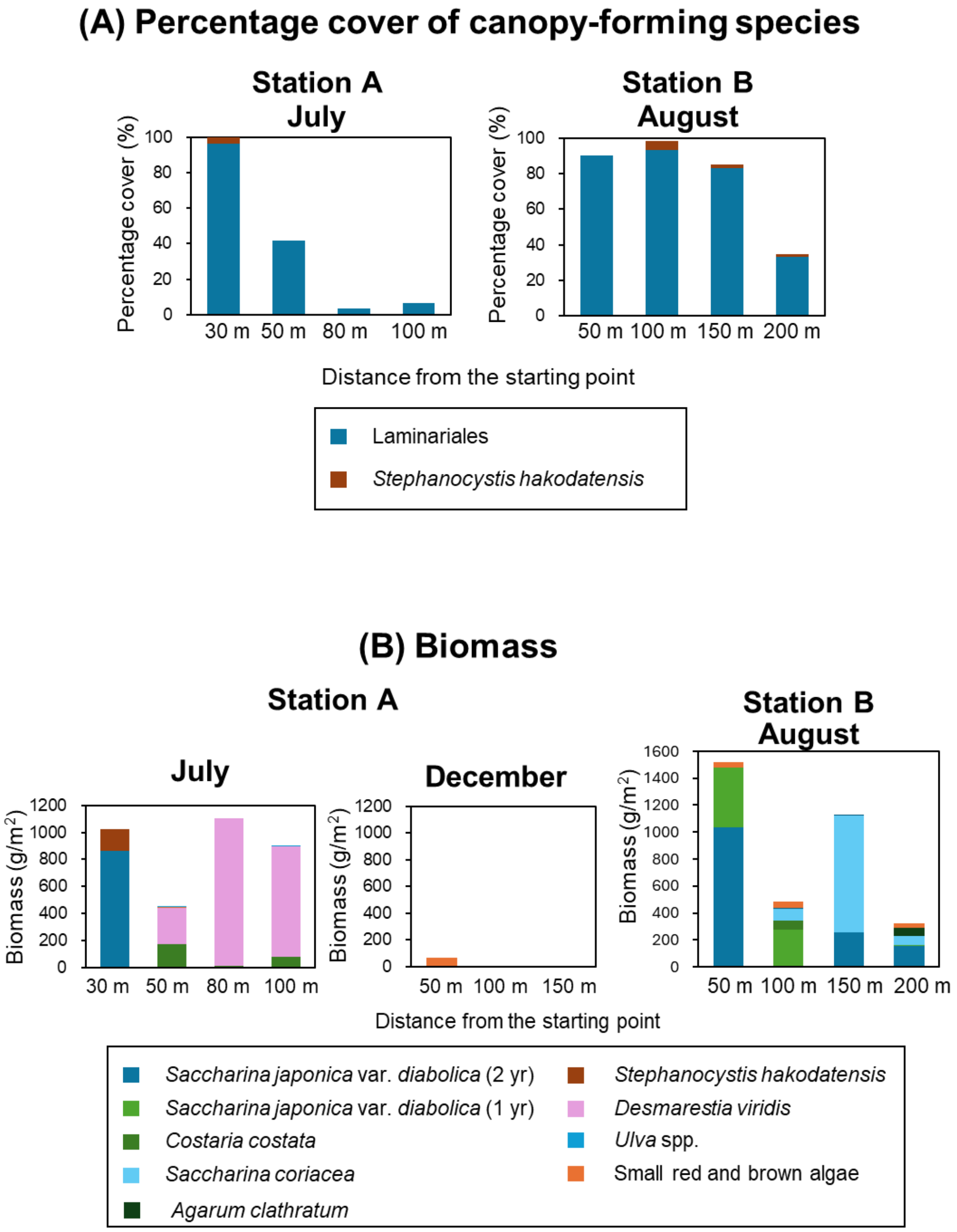

Figure S2. Percentage cover of canopy-forming species (A) and algal biomass (B) at sites A and B (n = 3). Vertical axes represent the distance from the starting point of the transects.

Table S1. Total number of sea urchins collected outer the transect lines.

Site	Month	Time for search	*Mesocentrotus nudus*	*Strongylocentrotus intermedius*
Station A	July	53 min		41
	December	20 min	2	43
Station B	August	7 min	7	3
